# A systematic review of high impact CpG sites and regions for MGMT methylation in glioblastoma [A systematic review of MGMT methylation in GBM]

**DOI:** 10.1186/s12883-024-03605-3

**Published:** 2024-03-23

**Authors:** David Gibson, Anh Huan Vo, Hannah Lambing, Prithanjan Bhattacharya, Peggy Tahir, Farid F. Chehab, Nicholas Butowski

**Affiliations:** 1 Department of Neuro-Oncology, University of California, San Francisco, 400 Parnassus Ave, San Francisco, CA USA; 2 Department of Epidemiology and Biostatistics, University of California, San Francisco, San Francisco, CA USA; 3 University of California, San Francisco Library, San Francisco, CA USA; 4 Department of Bioinformatics, University of California, Los Angeles, Los Angeles, CA USA; 5 Department of Laboratory Medicine, University of California, San Francisco, San Francisco, CA USA; 6 Department of Human Genetics, University of California, Los Angeles, Los Angeles, CA USA

**Keywords:** MGMT, Methylation, Glioblastoma, CpG, Epigenetics

## Abstract

**Background:**

*MGMT *(O 6 -methylguanine-DNA methyltransferase) promoter methylation is a commonly assessed prognostic marker in glioblastoma (GBM). Epigenetic silencing of the *MGMT* gene by promoter methylation is associated with greater overall and progression free survival with alkylating agent regimens. To date, there is marked heterogeneity in how *MGMT* promoter methylation is tested and which CpG sites are interrogated.

**Methods:**

To further elucidate which *MGMT* promoter CpG sites are of greatest interest, we performed comprehensive searches in PubMed, Web of Science, and Embase and reviewed 2,925 article abstracts. We followed the GRADE scoring system to assess risk of bias and the quality of the studies we included.

**Results:**

We included articles on adult glioblastoma that examined significant sites or regions within *MGMT* promoter for the outcomes: overall survival, progression free survival, and/or MGMT expression. We excluded systemic reviews and articles on lower grade glioma. fifteen articles met inclusion criteria with variable overlap in laboratory and statistical methods employed, as well as CpG sites interrogated. Pyrosequencing or BeadChip arrays were the most popular methods utilized, and CpG sites between CpG’s 70–90 were most frequently investigated. Overall, there was moderate concordance between the CpG sites that the studies reported to be highly predictive of prognosis. Combinations or means of sites between CpG’s 73–89 were associated with improved OS and PFS. Six studies identified CpG sites associated with prognosis that were closer to the transcription start site: CpG’s 8, 19, 22, 25, 27, 32,38, and CpG sites 21–37, as well as low methylation level of the enhancer regions.

**Conclusion:**

The following systematic review details a comprehensive investigation of the current literature and highlights several potential key CpG sites that demonstrate significant association with OS, PFS, and MGMT expression. However, the relationship between extent of *MGMT* promoter methylation and survival may be non-linear and could be influenced by potential CpG hotspots, the extent of methylation at each CpG site, and MGMT enhancer methylation status. There were several limitations within the studies such as smaller sample sizes, variance between methylation testing methods, and differences in the various statistical methods to test for association to outcome. Further studies of high impact CpG sites in MGMT methylation is warranted.

**Supplementary Information:**

The online version contains supplementary material available at 10.1186/s12883-024-03605-3.

## Introduction

Glioblastoma (GBM) is the most common and aggressive malignant primary brain tumor among adults. Accounting for 50–60% of total glioma diagnoses, the median overall survival (OS) for a patient diagnosed with GBM is 14–16 months [1]. In 2021, the WHO classification of tumors of the central nervous system restricted the diagnosis of GBM to IDH-wild type astrocytic tumor and reclassified all IDH-mutant diffuse astrocytic tumors as CNS WHO 2–4 [2]. Standard course of therapy for GBM includes surgical resection, temozolomide (TMZ) chemotherapy, and concomitant radiotherapy [3]. Since temozolomide’s initial inclusion as a standard alkylating agent for GBM, the O^6^-methylguanine DNA methyltransferase (*MGMT*) gene has become a key molecular marker. Despite the mass adoption of *MGMT* testing for prognostic purposes, there is no definitive best method for assessing the marker. The *MGMT* gene is a highly conserved sequence located at 10q26.3 on chromosome 10. *MGMT* encodes for the DNA repair protein, O^6^-alkylguanine-DNA-alkyltransferase (MGMT), which protects against alkylation at the O^6^ position on guanine from alkylating agents such as TMZ by transferring the methyl group to an internal cystine residue [4, 5]. This repair process is instrumental in genomic stability as MGMT repairs and prevents errors during DNA replication and transcription [5, 6]. A single MGMT protein can only repair one alkyl adduct as the DNA alkyl group binds irreversibly to its cysteine residue, permanently inactivating itself while repairing the DNA. For this reason, the capacity to repair O^6^-alkylguanine corresponds directly to the amount and production rate of MGMT [7].

MGMT acts independent of target site chromatin remodeling, apart from when its promoter region is methylated [4, 8, 9]. Epigenetic silencing of the *MGMT* gene through methylation of CpG sites within the promoter is associated with decreased expression of MGMT. Reduction of MGMT protein production results in reduced guanine nucleotide repair capacity, rendering alkylating agents such as TMZ more effective. For this reason, *MGMT* promoter methylation is clinically and prognostically advantageous as patients who have methylated *MGMT* promoters are more sensitive to chemotherapy. Studies have constantly demonstrated that tumors with unmethylated *MGMT* promoters have less favorable progression free survival (PFS) and Overall Survival (OS) outcomes when compared to their methylated counterparts [4, 10, 11].

The *MGMT* promoter contains a 777-base pair (bp) CpG islands (CGI) with 97 individual CpG sites. Studies using GBM cell lines identified two large regions within the promoter that show differences in methylation levels [12, 13]. One of these regions is most commonly investigated by the commercially prevalent assay, methylation specific PCR (MSP). The MSP region includes nine CpG sites that partially cover the first noncoding exon and the minimal enhancer. It remains unclear whether these nine CpG sites best reflect the status of MGMT expression as conflicting results from another study determined that the changes in methylation between an MGMT­ expressing and non-expressing cell line are focused in four CpG sites rather than being diffusely and uniformly distributed throughout the CGI [14]. MSP is often utilized to report a samples methylation as a binary status (methylated/unmethylated) and while it is highly prevalent other methods have become popular.

Among the most prevalent methylation testing alternatives to MSP are BeadChip assays, pyrosequencing (PSQ), multiplex ligation-dependent probe amplification (MS-MLPA) and immunohistochemistry (IH). In pyrosequencing, after the DNA is isolated, it undergoes bisulfite treatment and PCR amplification, and then a sequencing-by-synthesis system is used to query methylation at each individual CpG site. PSQ is ideal for giving a quantitative measurement of methylation and can be combined with a threshold for methylation status dichotomization. In 2016, a study by Quillen et al. demonstrated that PSQ has strong interlaboratory reproducibility and can reliably investigate MGMT status [15]. Unlike PSQ, multiplex ligation-dependent probe amplification and immunohistochemistry, does not have a bisulfite conversion component. The high-throughput profiling of the DNA methylome using BeadChip arrays has gained prominence due to its capacity to analyze a substantial range of CpG sites, typically ranging from 450,000 to 850,000, with a high degree of accuracy and cost-effectiveness.

While the debate over the objectively best method to measure *MGMT* methylation will continue to evolve as technology improves, it is important to discern which CpG sites or combinations of CpG sites within the CGI have the greatest impact on expression and survival. The breadth of knowledge on this topic is sparse despite its potential clinical implications in precision medicine. This current systematic review aims to compile and review the findings of relevant research to better understand the differential prognostic impact of silencing certain CpG sites within the *MGMT* CGI. After careful review of available literature, we present the results of 15 publications aimed at informing researchers of potential *MGMT* promoter CpG sites of interest in GBM.

## Methods

### Search methods

**Fig. 1:**
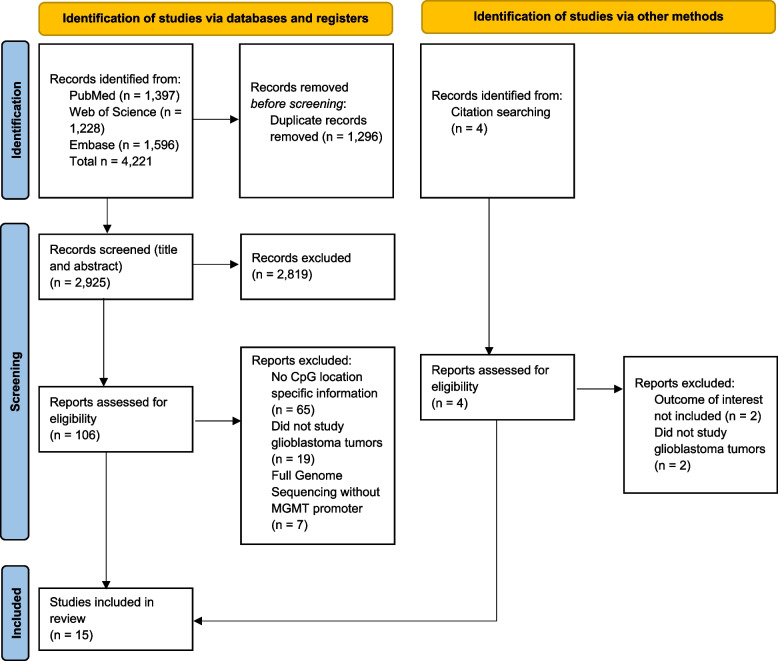
Literature search and screening plan. *From: *Page MJ, McKenzie JE, Bossuyt PM, Boutron I, Hoffmann TC, Mulrow CD, et al. The PRISMA 2020 statement: an updated guideline for reporting systematic reviews. BMJ 2021;372:n71. doi: 10.1136/bmj.n71. For more information, visit: http://www.prisma-statement.org/

We performed comprehensive searches in PubMed, Web of Science, and Embase to find relevant articles; searches were conducted on12/21/2023, and all articles inclusive to that date were included (Fig. 1). We searched broadly across four main concepts: glioblastoma, DNA methylation, *MGMT* promoter, and overall survival. Multiple synonyms were developed across concepts to ensure sensitivity and not miss any important articles. We used both index terms (Mesh and Emtree) and keywords in the searches. Full search strategies for each database are included in the Additional file 1. Gray literature was investigated by hand-searching the references for papers included for data extraction and reviewing conference abstracts from Embase for trends and background information, or potential unpublished articles. This study utilized Zotero software to manage references while performing review. R and Adobe illustrator were used to visually display results of studies.

### Study selection

All titles and abstracts of references compiled during the initial database search were independently screened and accepted if the article met the following inclusion criteria: (1) The study must have been on adult glioblastoma primary tumors; (2) The study’s objective was determining significant sites or regions within *MGMT* promoter for the outcomes: OS, PFS, and/or MGMT expression; (3) for OS and PFS outcomes, study subjects underwent surgical resection followed by treatment with TMZ and radiotherapy. Articles and abstracts were excluded if they were a systematic review, a case study, only on lower grade gliomas, or reported on the entire methylome with no results in the *MGMT* promoter region. The outcomes of the studies we screened, and their effect measures were progression free survival and overall survival by hazard ratio, as well as MGMT expression via concordance.

### Risk of bias

To Assess risk bias and the quality of the studies we included, we followed the GRADE (Grading of Recommendations Assessment, Development, and Evaluation) scoring system. The GRADE scoring system assesses the study design, statistical methods, publication bias, effect sizes, dose response, and residual confounding. Using the GRADE system, we assessed the quality and certainty of the evidence presented by the articles included in this study. Evidence was scored as either high, moderate, low, or very low. We also used comprehensive search terms and the reviewers worked independently in order to minimize reporting bias.

## Results

### Search and selection process

Article search was carried out using PubMed, Web of Science, and Embase resulting in 1397, 1228, and 1596 references respectively (Fig. 1). After removing duplicates from our compiled reference list, we had 2925 references. Following an initial abstract review of all compiled references, inter-rater cross reference comparison resulted in 106 reference abstracts that met our inclusion/exclusion criteria. Further review of the complete manuscripts for the 106 accepted references and additional hand searched articles in these references resulted in a final count of fifteen publications included in this systematic review. Articles were excluded during the search for lack of detail on which specific CpG sites or combinations of CpG sites in the *MGMT* CGI were associated with OS, PFS, or MGMT expression. Characteristics of the studies that were included from the search can be found in Table 1.

**Table 1:** Table of characteristics for studies included in this systematic review

Authors	Reference #	GRADE	Year of Initial Publication	Subjects (Samples)	Tissue	Main lab method	Statistical Method	Outcome	Survival data?	IDH status included	CpG Type	Number of CpG sites Interrogated	List of CpG sites Interrogated	CpG sites of interest (associated with outcome)^a^
Yildiz et al.	[16]	Low	2007	100 (100)	FFPE	BS, PR, PSQ	χ², K-M, log-rank test, Cox PH	OS, PFS	Yes	No	Islands	4 islands	CpG1, CpG2, CpG3, and CpG4	PFS: CpG3; none found significant for OS
Everhard et al.	[14]	Moderate	2009	78 (54 GBM)	Frozen	PSQ, Expression Analysis, PCR	Mann-Whitney test, hierarchical clustering	Expression	Yes	No	Sites, Regions	68	1-87	Correlated with expression: 27, 32, 73, 75, 79 and 80; Concordant with expression: 32-33, 72-83
Malley et al.	[17]	High	2011	22 (41)	Xenograft	Luciferase assay, PSQ, RT-PCR	Unsupervised hierarchical clustering, Mann-	mRNA expression, luciferase	No	No	Island, Cluster	96	Entire MGMT CGI	83, 86, 87, 89
Shah et al.	[18]	High	2011	70 (gene expression=4 6, PFS=44, IHC=31)	Frozen	BS, PCR, IHC, MS-MLPA	3R classification, Spearman's rank correlation	mRNA expression, protein expression, and PFS	Yes	No	Sites, regions	97	Entire MGMT CGI	PFS: 8,22,38^c^,73^c^,75- 78,80- 82^c^,86^c^,87,88,89^c^
Bady et al.	[19]	Moderate	2012	63 (59 GBM)	Frozen	BS, HM-450K, HM-27K	Univariate model and stepwise logistic model building	OS	Yes	Yes	Sites	176 (14 in promoter)	17-20, 22, 25, 31, 60-62, 64, 70, 84, 97	19, 25
Quillien et al.	[15]	Moderate	2014	89 (89)	FFPE	PSQ	Cox - AUROC, KM, bootstrapping Harold-C index	OS, PFS	Yes	No	Sites	16	74-83, 84-89	89, means of 84- 88, 85-89, and 74- 89
De Carlo et al.	[20]	Low	2015	121 (121)	NA	PSQ	Uni and multivariate Cox regression	OS, PFS	Yes	No	Sites	10	74-83	74-77, 79-83
Mur et al.	[21]	Very low	2015	68 (68)	Frozen/PE	BS, MSP, qMSP, HM- 450K	PCA and unsupervised hierarchical clustering, K-M, log- rank test, Spearman's correlation coefficient, multivariable Cox PH	OS	Yes	Yes	Sites	154 (25 in promoter)	1-10, 13-27	25, 165
Chai et al.	[1]	Moderate	2018	159 (82^b^,77^b^)	FFPE	RNA-seq, PSQ	Spearman's correlation analysis, ROC	mRNA expression, OS	Yes	Yes	Sites, Combination	11	75-82 (cohort #1), 72- 78(cohort #2)	76-79 and 74-78
Siller et al	[22]	Low	2021	215 IDH wild type	FFPE	MSP, Sanger	K-M, Log-rank test, Cox regression models, Multiple proportional hazards models, Spearman and Pearson correlation coefficient testing	OS	Yes	Yes	Sites	25	74–98	74–98
Caccese	[23]	High	2022	591 IDH wild type	FFPE	PSQ	Mann–Whitney U test ,χ², K-M, Cox regression model	OS	Yes	Yes	Sites	10	75–84	75–84
Leske et al.	[24]	Low	2023	32 long term survivors and 25 short term survivors	FFPE/Frozen	BS, PCR, Sanger sequencing	Random Forest regressions, conditional Random Forest analysis	OS	Yes	Yes	Sites	79	CpG 23 (bp − 300) to the end of the CpG island (bp + 289), CpG sites (bp + 292, + 296, + 309	86 has the highest predictive value. 75, 77, 78, 84, 88, and 96
Buyuktepe et al.	[25]	Moderate	2023	95 (34 IDH wildtype)	FFPE	NGS, BS, MS- PCR, MS-HRM	Shapiro-Wilk test, t- test/Mann Whitney U, χ²/Fisher's exact test, K-M, Cox -ratio hazard model	OS/PFS	Yes	Yes	Islands/ whole promoter	32 and whole promoter	CpG 70–78, CpG 79–83, CpG 84–87, CpG 70–87, and whole promoter	OS and PFS: CpG 79–83 and CpG 84–87
Gibson et al.	[26]	Low	2023	300 (5 IDH mutant)	FFPE	MSP, CLIA based assay and BS	Cox proportional hazard,χ²	OS/PFS	Yes	Yes	Sites	17	21-37	medium levels of promoter methylation had greatest reduction in HR
Zappe et al.	[27]	Low	2023	40 GBM (1 IDH mutant, 1 gliosarcoma)	FFPE/T98G cell line	PCR, HRM, PSQ	One-way ANOVA followed by a post hoc t-test, K-M, scatter plot and Pearson's correlation coefficient	OS/MGMT protein level	Yes	Yes	Sites/Enhancer regions	61	CpG: 72-83, 4 enhancer regions	promoter: 75,78,80. enhancer region: low methylation of 01–03 and 09–13

χ² Chi-squared test

*K-M* Kaplan Meier method, *Cox PH* Cox proportional hazard

^a^If outcome not specified, then CpG sites of interest correspond to all outcomes listed

^b^Samples were split into two cohorts: cohort #1 and cohort #2

^c^Associated with mRNA expression and MGMT protein expression, in addition to PFS

### Study descriptions and outcomes

**Fig. 2:**
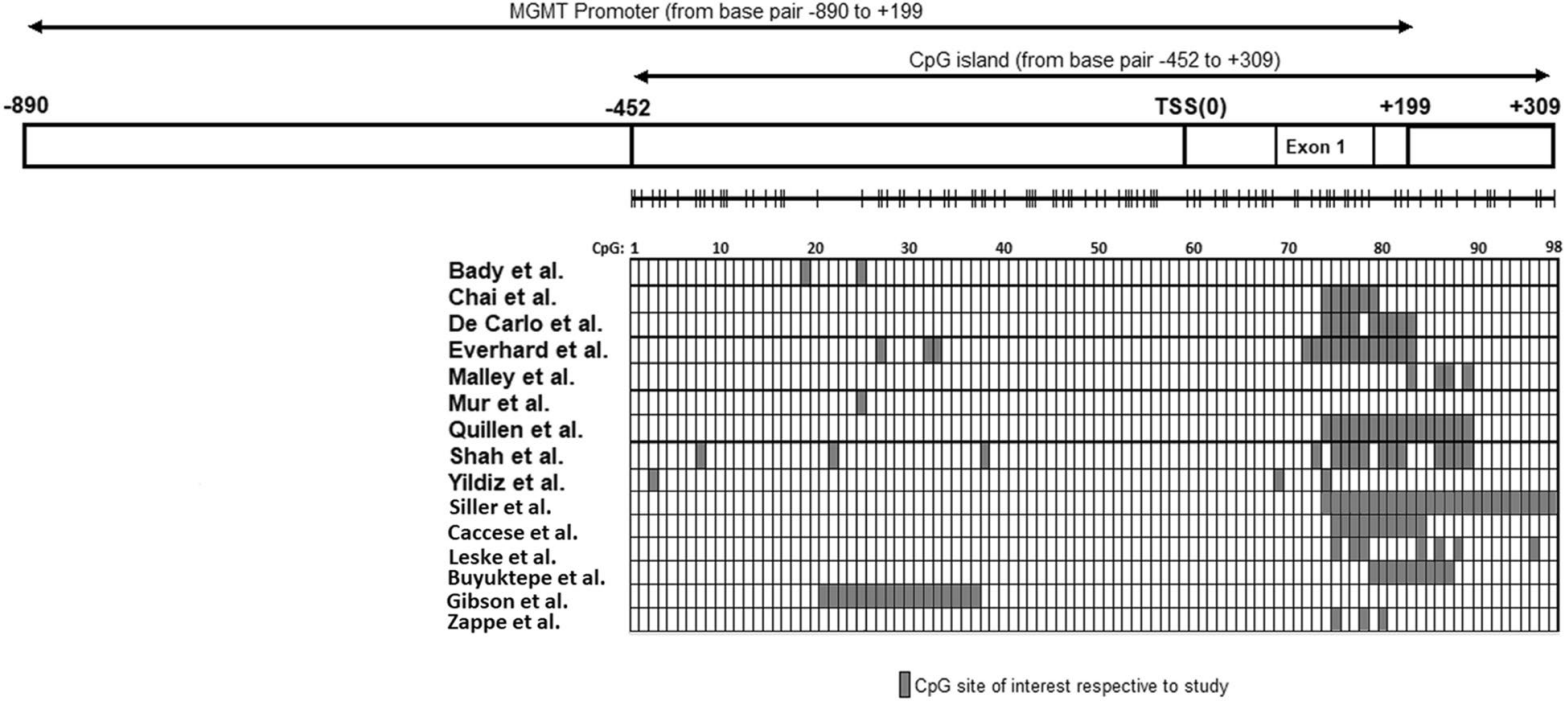
Illustration of the MGMT promoter and the location of CpG sites, that were associated with outcomes, interrogated by the various studies included in this systematic review

The following studies were included. An exhaustive list of the methods used by each study can be found in Table 1 and a comprehensive illustration of the MGMT CGI and the CpG sites that were interrogated by each study can be found in Fig. 2. The study by Malley et al., which has high GRADE scoring, investigated how individual CpG sites in the *MGMT* CGI impacted transcriptional control and aimed aim to define the region suitable for clinical *MGMT* methylation testing [17]. The study investigated the methylation status of the entire *MGMT* promoter CGI using pyrosequencing and compared it with *MGMT* mRNA expression in a series of glioblastoma cell lines and xenografts. They identified two separate differentially methylated regions (DMR1 and DMR2), CpG25-50 and CPG73-90, where methylation status was significantly correlated with expression. Interestingly, DMR1 and DMR2 did not show the highest mean methylation levels within the entire *MGMT* CGI suggesting that not all methylated CpG sites are directly involved in transcriptional regulation. Further investigation using a luciferase reporter assay suggested that several CpG sites within DMR2 is critical for transcriptional control. CpG sites 83, 86, 87 and 89 within DMR2 seemed to play a critical role in transcriptional control of *MGMT*. The reporter assay that was used demonstrated that mutations of either the individual CpG or multiple consecutive CpG sites significantly attenuated promoter activity. In particular, mutation of CpG 89 alone almost completely abolished the promoter activity.

Everhard et al., which has moderate GRADE Scoring, studied methylation at 68 CPG sites within the *MGMT* CGI by performing pyrosequencing on 54 GBM tumors and 24 normal brain samples [14]. Methylation levels were compared with expression in 52 out of the 68 CpG sites where methylation was found to only be observed in the tumor samples. There was considerable overlap in Everhard et al. and Malley et al.’s findings as they found that methylation of CpGs 27, 32, 73, 75, 79 and 80 were most significantly correlated with expression, and that methylation status at CpGs 32–33 and 72–83 was most concordant with expression. All the aforementioned sites by Everhard et al. fall into the two differentially methylated regions (DMR) defined in Malley, et al.’s study. A potential criticism of Everhard et al.’s approach is that assessment of mRNA expression in primary tumor tissues may have been hampered by the presence of non-neoplastic cells in the tissue which may express MGMT. This study also determined that the region most commonly assessed by MSP was not among the regions determined to be correlated with expression.

In another study utilizing pyrosequencing to assess CpG site methylation, Chai et al., which has moderate GRADE scoring, evaluated 51 GBM patients out of a cohort of 223 Chinese Glioma Genome Atlas patients [1]. They tested the prognostic effect of methylation on several different combinations of 11 different CpG sites (72–82) by comparing the area under the curve of ROC’s for the various different combinations. The study concluded that CpG combinations with four or more CpGs from CpGs 72–82 are enough to eliminate the influence of heterogeneity among individual CpG sites. Following this finding, they concluded that CpG combinations with four or more consecutive CpGs within CpGs 72–82, including the combinations of CpGs 76–79 and CpGs 74–78 used in commercial kits, are equally effective at predicting *MGMT* mRNA expression and the survival of TMZ treated glioma patients. Their results also suggested that testing combinations with part of CpGs within CpGs 76–79 or CpGs 74–78 presented poorer predictive value than when the clusters were included entirely. Lastly, for the commonly used combinations of CpGs 76–79 and CpGs 74–78, it is suggested to analyze the entire methylation status by including all CpG sites within 72–82. Despite these significant findings, this study was limited by a rather small GBM sample size and small number of *MGMT* promoter CpG’s tested (11/97).

In 2011, Shah, et al., which has high GRADE scoring, published a comprehensive analysis on *MGMT* promoter methylation and its correlation with both MGMT expression and clinical response [21]. The study used an array of methods including bisulfite sequencing, methylation-specific multiplex ligation-dependent probe amplification (MS-MLPA), immunohistochemistry (IHC), and polymerase chain reaction (PCR) on the entire *MGMT* CGI on 70 GBM patient samples. Bisulfite sequencing revealed that that the methylation profiles of the various samples were highly heterogeneous. By performing hierarchical clustering of methylation patters among all samples they identified three major CpG site clusters with the coordinates for each region being measured from the transcription start site (TSS). Region 1 (R1) coordinates are − 452 to − 317 (15 CpGs), Region 2 (R2) coordinates are − 302 to − 105 (29 CpGs), and Region 3 (R3) coordinates are + 40 to + 255 (27 CpG sites). Using this data, they proposed a novel classification method termed “3R” which utilized methylation data from across the entire promoter and grouped patients based on which regions were methylated. To adapt this approach to the clinical setting and to test its predictive ability for PFS, they employed an MS-MLPA test based on the 3R classification. The authors determined that CpG sites 8, 22, 38, 73, 75, 76, 77, 78, 80, 81, 82, 86, 87, 88, and 89 were predictive for improved PFS and that sites 38, 73, 80, 81, 82, 86, and 89 were correlated with mRNA expression, MGMT protein expression, and improved PFS.

The study by Mur, et al. was another instance of whole *MGMT* CGI methylation data being acquired by means of Illumina’s HM-450k BeadChip platform [19]. However, this study has very low GRADE scoring. Whole-genome methylation profiles were obtained from 247 samples. The vast majority of the analysis examined the effect of individual CpG sites within all gliomas but there was a sub-analysis that was specifically in TMZ treated GBM patients. The study concluded that there was a lack of association between CpG 25 and OS. While gene body CpG 165 was associated with improved prognosis is some of the other glioma classifications they assessed, they determined there was no significant association with OS in GBM patients.

In a study that builds upon the findings of Shah et al., Bady et al., which has moderate scoring, also utilized the HM-450 K BeadChip to interrogate 176 CpGs annotated for the MGMT gene, 14 of which were located in the promoter [20]. This study combined 4 external data sets with their own data on 63 GBM tissues from 59 patients to arrive at a sample size of 352 GBM methylation profiles. Their findings coincided with those of Malley et al. where they identified two distinct regions, DMR1 and DMR2, which demonstrated high importance for gene silencing and outcome prediction. The study also found a strong association between elven probes located in the TSS-encompassing CpG island of the MGMT promoter were significantly associated with previously MSP-defined MGMT methylation status. However, the strongest association was reached by probes for CpG’s 19 and 25.

The 2014 study conducted by Quillien et al., studied a cohort of 89 newly diagnosed adult GBM patients to determine which individual CpG, or combination of CpGs is best at predicting therapeutic response [15]. This study has moderate GRADE scoring. Using PSQ, this study interrogated 16 CpGs (CpGs 74–89) and determined their impact on PFS and OS by means of ROC (Cox PH) and post bootstrapping Harold C-index. Among the topmost ten ranking GpGs or means of CpGs associated with outcome, Quillien et al. determined that CpG 89, means of CpGs 84–88, 85–89 and 74–89 was most strongly associated with improved prognosis. Interestingly, some of the samples determined to be heterogeneously methylated via PSQ were found to be unmethylated when tested with MS-PCR. The authors concluded with a recommendation to test CpGs 74–78 using PSQ with the PyroMark CpG MGMT kit and to use the mean score to determine methylation status due to the kits commercial availability.

The study by De Carlo et al. was the only conference abstract included in this systematic review [16]. This study has low GRADE scoring. De carlo e al. analyzed 10 CpG sites (74–83) of 121 GBM patients by means of PSQ. Univariate analysis found all CpG sites apart from 78 to be statistically significant for OS and PFS. Reported in a table included in the abstract, but not directly stated, CpG’s 74, 75, 80, and 81 displayed the greatest reduction in hazard.

The study by Yildiz et al., which has low GRADE scoring, was a Turkish study that examined the prognostic significance of *MGMT* methylation at 4 CpG islands [18]. However, the author does not specify the location of these “CpG islands” apart from them being located within the *MGMT* promoter. For this reason, we find the following results to be rather uninformative. The author concluded that among patients treated with radiation therapy and TMZ, methylation status of CpG1, CpG2, CpG3 and CpG4 islands had no effect on OS or PFS. For those who only underwent radiation therapy, methylation of the four islands had significant effects on PFS. Overall, methylation of the CpG3 island was a good prognostic predictor for a positive effect on PFS.

There are six studies included that were published after the 2021 WHO classification of tumors of the central nervous system, which defined GBM as IDH-wild type astrocytic tumors. Siller et al., which has low GRADE scoring, described a large cohort of 215 IDH-wild type GBM tumors’ *MGMT* status using MSP and Sanger sequencing [22]. They examined 25 CpG sites from CpGs 74–98 in the DMR-2 island and downstream. In the study, there was neighborhood-dependent propagation of methylation of the *MGMT* promoter. The authors also found that *MGMT* methylation at all 25 sites correlated with survival, and survival was linearly associated with cumulative numbers of methylated CpG sites, especially in patient who received TMZ. There was no identified hot-spot as all examined CpG sites contributed equally to the effect. However, subsequent studies found that the relationship between the extent of MGMT promoter methylation and survival in GBM may be nonlinear [23, 26]. This suggests that a simple cut off for MGMT promoter status might be inadequate for prognosis and further quantitative and detailed examination of individual CpG sites are warranted [26].

The 2022 study, conducted by Caccese et al., which has high GRADE scoring, investigated *MGMT* methylation status and its association with OS in a multicenter study [23]. The study included 591 patients with IDH-wildtype GBM and *MGMT* status was examined using quantitative PSQ method. The authors examined CpG sites from 75 to 84 and suggested a cut-off of 15% for *MGMT* methylation status regarding survival. They also noted significant variations in cut-off % used for methylation status analyzed with PSQ in previous studies ranging from 5 to 29%. The authors concluded that the relationship between MGMT promoter methylation and survival may be non-linear as there is slightly decreased OS in patients with > 40% MGMT promoter methylation.

 Additionally, a recent study in 2023 by Gibson et al., which has low GRADE scoring, looked at 17 specific CpG sites and found a non-linear relationship between extent of *MGMT* promoter methylation and survival in GBM [26]. The authors of this study examined the total numbers of methylated *MGMT* promoter CpG sites in 17 different locations (CpGs 1–5 are located 85 bp upstream of DMR1 and CpGs 6–17 extended 106 bp into DMR1) using MSP and CLIA-based assay and bisulfite sequencing techniques in 300 GBM patients. This correlates to CpG sites 21–37 on the *MGMT* promoter gene. Of note, there were 15 IDH-mutant astrocytoma patients included as GBM in this study. They found that while patients with low level of methylation (1–6 CpG sites) fared the worst, patients with medium level of methylation (7–12 CpG sites) did better than high level of methylation (13–17) regarding survival. This study did not take into account how heavily methylated each CpG site is and if there is CpG subgroups with superior impact regarding survival. Another limitation of this study is that while previous studies demonstrated that the DMR1 region is important for transcription silencing, the authors interrogated CpG sites closer to the transcription start site whereas most recent studies investigated CpG sites 70–90.

More recently, a study by Leske et al., which has low GRADE scoring, looked into methylation patterns of specific *MGMT* CpG sites in IDH-wild type GBM in 32 long-term survivors (OS of more than 3 years) and short-term survivors (OS of less than 1 year) [24]. They employed bisulfite converted DNA using MSP followed by Sanger sequencing to interrogate 79 CpG sites. They found that long-term survivors often had CpGs peak at 28–40 and 75–96. In this study, random forest analysis demonstrated that CpG 86 had the highest predictive value for OS. The authors concluded that further examination of this MGMT CpG region is warranted. Other CpG sites such as 75, 77, 78, 84, 88, and 96, also had strong predictor importance for survival. A potential drawback of this study is that patients with survival between one and three years were not included, and thus missing data might influence the outcome.

Similarly, a study by Buyuktepe et al., which has moderate GRADE scoring, used next generation sequencing assay in 95 GBM patients to investigate the most predictive CpG islands within *MGMT* promoter and its association with OS and PFS [25]. They employed a 10% cut off for methylation and grouped result into CpG islands of CpG 70–78. CpG 79–83, CpG 84–87, CpG 70–87, and whole promoter. Their result overlapped with the those of Leske et al., and the studies reported in this review. The authors found that methylation of CpG islands 79–83 and 84–87 is associated with better survival outcome [25]. A significant limitation of this study is that 34 patients had IDH-mutant GBM, which has improved survival outcome compared to IDH-wildtype.

Finally, a study by Zappe et al., which has low GRADE scoring, evaluated both *MGMT* enhancer and *MGMT* promoter methylation association with survival and MGMT protein expression in GBM [27]. *MGMT* promoter methylation status of CpGs 72–83 was evaluated using PSQ in 38 patients with GBM (one patient had IDH-mutant GBM, and one patient had gliosarcoma). In patients with IDH-wildtype GBM, methylated *MGMT* promoter had significant higher OS, and methylation levels of CpGs 75, 78, and 80 correlated positively with OS. However, they also hypothesized that methylation of *MGMT* enhancers also contributes to MGMT production and potentially survival. Using their in-house developed methylation analysis methods, the authors found that low methylation of CpGs 01–03 and 09–13 in enhancer 4 region of *MGMT* is associated with favorable OS in GBM patients. Methylation of enhancer 2 (560 kb upstream of the *MGMT* promoter) and enhancer 3 (CpGs 15–22) is also negatively associated with MGMT protein expression.

## Discussion

In this systematic review we reviewed the methods and results of fifteen different studies that aimed at discerning which CpG sites located within the MGMT CGI offered varying degrees of effect on OS, PFS, and expression. There were varying degrees of overlap in the laboratory and statistical methods employed by the various studies included in this systematic review. There was also a considerable amount of overlap in the CpG sites that were tested. Many of the studies included utilized PSQ and interrogated CpG sites between CpG 70 and CpG 90 [1, 14, 16, 17, 22–25, 27]. Other studies however employed more comprehensive approaches by using either a combination of methods, BeadChips that were capable of interrogating the entire CGI, or publicly available data acquired by these methods [15, 19–21].

There is variable degree of methylation at each individual CpG sites in all *MGMT* promoter methylation assays [28]. Thus, it is challenging to calculate the methylation percentage of a specific CpG site. For this reason, we opted not to include the percentage of methylation cut off at CpG sites. There was moderate concordance between the CpG sites that the various studies reported to be highly predictive of outcome. Many of the studies reported that specific CpG sites and different combinations/means of sites between CpGs 73–89 were associated with improved OS and PFS. The relationship between extent of *MGMT* promoter methylation and survival may be non-linear and could be influenced by potential CpG hotspots, the extent of methylation at each CpG site, and MGMT enhancer methylation status. Five studies identified CpG sites associated with prognosis that were located earlier on in the CGI, closer to the transcription start site [14, 19–21, 26]. Collectively, these five studies identified CpG’s 8, 19, 22, 25, 27, 32, 38, and CpG sites 21–37, yet no two studies identified the same CpG to be predictive with prognosis. Lastly, a study looking at *MGMT* enhancer using their own in-house analysis method found that low methylation of the enhancer regions 2,3, and 4 can also influence survival and MGMT protein production.

There were several limitations within the studies that we identified that may lead to bias or disparity between predicted measure of effect and true measure of effect. Many of these studies had smaller sample sizes, with some using publicly available methylation data to increase their study population [16, 18, 19]. Apart from studies that employed BeadChip arrays, most studies interrogated select CpG sites within the *MGMT* CGI, mostly due to the commercial availability of certain PSQ kits. The studies that employed BeadChip added subjects from various public databases to bolster sample sizes [19, 20]. In addition to variance between methylation testing methods, there were also differences in the various statistical methods to test for association to outcome. Lastly, all of these studies had both left censoring, where the amount of time a patient was living with GBM before getting diagnosed was unknown, and right censoring, where there was loss to follow up or the study ended prior to the patient passing away.

 Apart from methodological differences there are also biological hurdles one faces when assessing the results of methylation testing and associating it with prognosis. GBM tumors are heterogenous, in which samples from the same tumor are often differentially methylated [29]. Not only might there be variance in the number of CpG sites methylated in a single tumor, there may also be variance in which CpG sites are methylated. Additionally, recent research has demonstrated that the basal level of MGMT expression is largely determined by the transcription factor SP1 [30]. Deletion of SP1 has been shown to exhibit a 200-400-fold decrease in the basic promoter activity in glioma cells [30]. Moreover, knockdown of SP1 strongly reduces MGMT protein expression, further suggesting SP1’s role as a main regulating factor for MGMT expression in glioma cells. With various biological mechanisms affecting MGMT expression, it is difficult to discern what is really driving down MGMT expression. Furthermore, a limitation of our search method is we did not differentiate the significance of *MGMT* methylation between IDH-mutant and IDH-wildtype astrocytic tumors. IDH status was included in 9 out of 15 studies in our review as shown in Table 1, 6 studies were published after the 2021 WHO classification of tumors of the central nervous system. These studies indicated that IDH mutations are strongly correlated with *MGMT* methylation status in GBM [1, 19, 20]. A study by Molenaar et al. showed that the combination of IDH1 mutation and *MGMT* methylation outperforms either IDH1 mutation or methylation status alone in predicting survival for GBM patients [31]. This comparison might be helpful in future research.

The evidence presented in this systematic review highlights several potential key CpG sites that demonstrate significant association with OS, PFS, and MGMT expression. More importantly, this study calls attention to the limited amount of research conducted in this area and the lack of concordance between the findings of some of the results. As greater laboratory and computational methods arise for methylome sequencing, it is essential more research is performed that directly investigates which CpG sites or combinations of sites offer the greatest prognostic advantage for glioblastoma patients.

### Supplementary Information

**Supplementary Material 1.**

## Data Availability

The authors confirm that the data supporting the findings of this study are available within the article [and/or] its supplementary materials.

## Abbreviations

BS: Bisulfite Sequencing
CGI: Cytosine and Guanine Island
CPG: Cytosine paired Guanine
FFPE: Formalin-Fixed Paraffin-Embedded
HC: Hierarchical Clustering
IHC: Immunohistochemistry
MS-MLPA: Methylation Specific Multiplex Ligation Dependent Probe Amplification
MSP: Methylation Specific PCR
NA: Not Available
PCA: Principal Component Analysis
PSQ: Pyrosequencing
RT-PCR: Real Time Polymerase Chain Reaction
