# Supplementary material for: A systematic review of high impact CpG sites and regions for MGMT methylation in glioblastoma [A systematic review of MGMT methylation in GBM]
Source: BMC Neurol. 2024 Mar 23;24:103. doi: 10.1186/s12883-024-03605-3 (PMC10960428; doi:10.1186/s12883-024-03605-3)
Supplement: Supplementary file 1 — Supplementary Material 1. [file 12883_2024_3605_MOESM1_ESM.docx]

**Search Appendix**

| DATABASE | SEARCH STRATEGY |
| --- | --- |
|  |  |
| PubMed | (“Glioblastoma”[mesh] OR glioblastoma) AND (“DNA Methylation”[mesh] OR methylation OR “DNA Modification Methylases/genetics”[Mesh] OR "Tumor Suppressor Proteins/genetics"[Mesh] OR "DNA Repair Enzymes/genetics”[Mesh] OR “DNA modification methylases” OR “tumor suppressor proteins” OR “DNA repair enzymes” OR “Bisulfite sequencing” OR "CpG Islands"[Mesh] OR “CpG islands”) AND ("Promoter Regions, Genetic"[Mesh] OR MGMT promoter OR methyl-guanine methyl transferase gene promoter OR “Biomarkers”[mesh] OR biomarker OR biomarkers OR continuous OR binary) AND (“Prognosis”[mesh] OR prognosis OR "Progression-Free Survival"[Mesh] OR “progression-free survival” OR “overall survival” OR "Survival Analysis"[Mesh] OR “survival analysis” OR “survival analyses” OR "Proportional Hazards Models"[Mesh] OR “Proportional Hazard” OR “Cox model”) |
| Web of Science | glioblastoma AND (methylation OR “DNA modification methylases” OR “tumor suppressor proteins” OR “DNA repair enzymes” OR “Bisulfite sequencing” OR “CpG islands”) AND (MGMT promoter OR methyl-guanine methyl transferase gene promoter OR biomarker OR biomarkers OR continuous OR binary) AND (prognosis OR “progression-free survival” OR “overall survival” OR “survival analysis” OR “survival analyses” OR “Proportional Hazard” OR “Cox model”) AND (prognosis OR “progression-free survival” OR “overall survival” OR “survival analysis” OR “survival analyses” OR “Proportional Hazards Model” OR “Cox model”) |
| Embase | ('glioblastoma'/exp OR glioblastoma) AND ('dna methylation'/exp OR 'dna methylation' OR 'methylation'/exp OR methylation OR 'dna methyltransferase'/exp OR 'dna methyltransferase' OR 'tumor suppressor protein'/exp OR 'tumor suppressor protein' OR 'dna ligase'/exp OR 'dna ligase' OR 'bisulfite sequencing'/exp OR 'bisulfite sequencing' OR 'cpg island'/exp OR 'cpg island') AND ('mgmt promoter' OR 'methyl-guanine methyl transferase gene promoter' OR 'biological marker'/exp OR 'biological marker' OR continuous OR binary OR ('methyl guanine' AND ('methyl'/exp OR methyl) AND ('transferase'/exp OR transferase) AND ('gene'/exp OR gene) AND ('promoter'/exp OR promoter))) AND ('prognosis'/exp OR prognosis OR 'progression-free survival'/exp OR 'progression-free survival' OR 'overall survival'/exp OR 'overall survival' OR 'survival analysis'/exp OR 'survival analysis' OR 'survival analyses' OR 'proportional hazards model'/exp OR 'proportional hazards model' OR ‘cox model’) |
